# Supplementary material for: Insights into the viral landscape of the western honey bee and native bees in Bangladesh
Source: Microbiol Spectr. 2025 Nov 11;13(12):e01971-25. doi: 10.1128/spectrum.01971-25 (PMC12671125; doi:10.1128/spectrum.01971-25)
Supplement: Supplemental figures — Figures S1 to S3. [file spectrum.01971-25-s0003.docx]

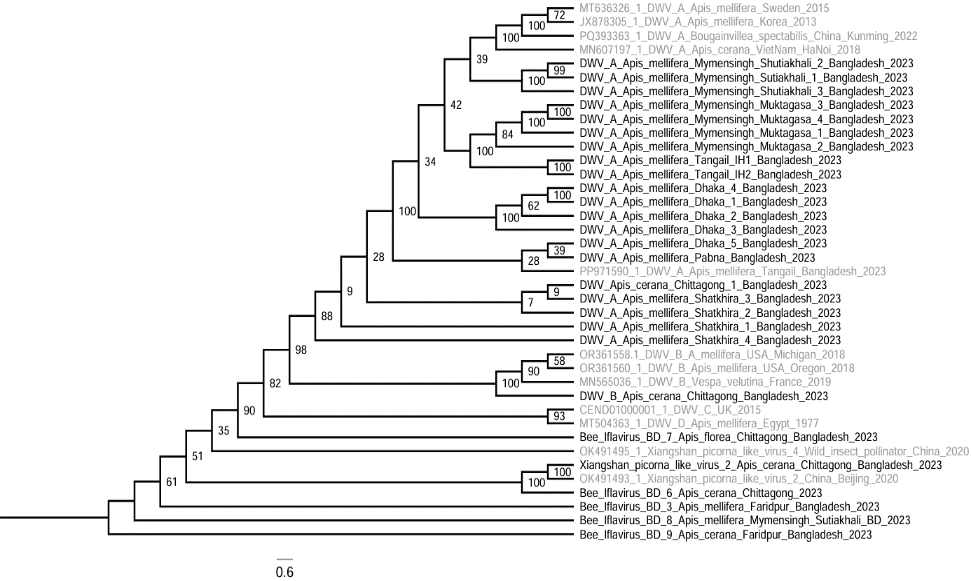


Supplementary figure 1. Phylogenetic analyses of DWV and potential outgroups of bee species in Bangladesh.


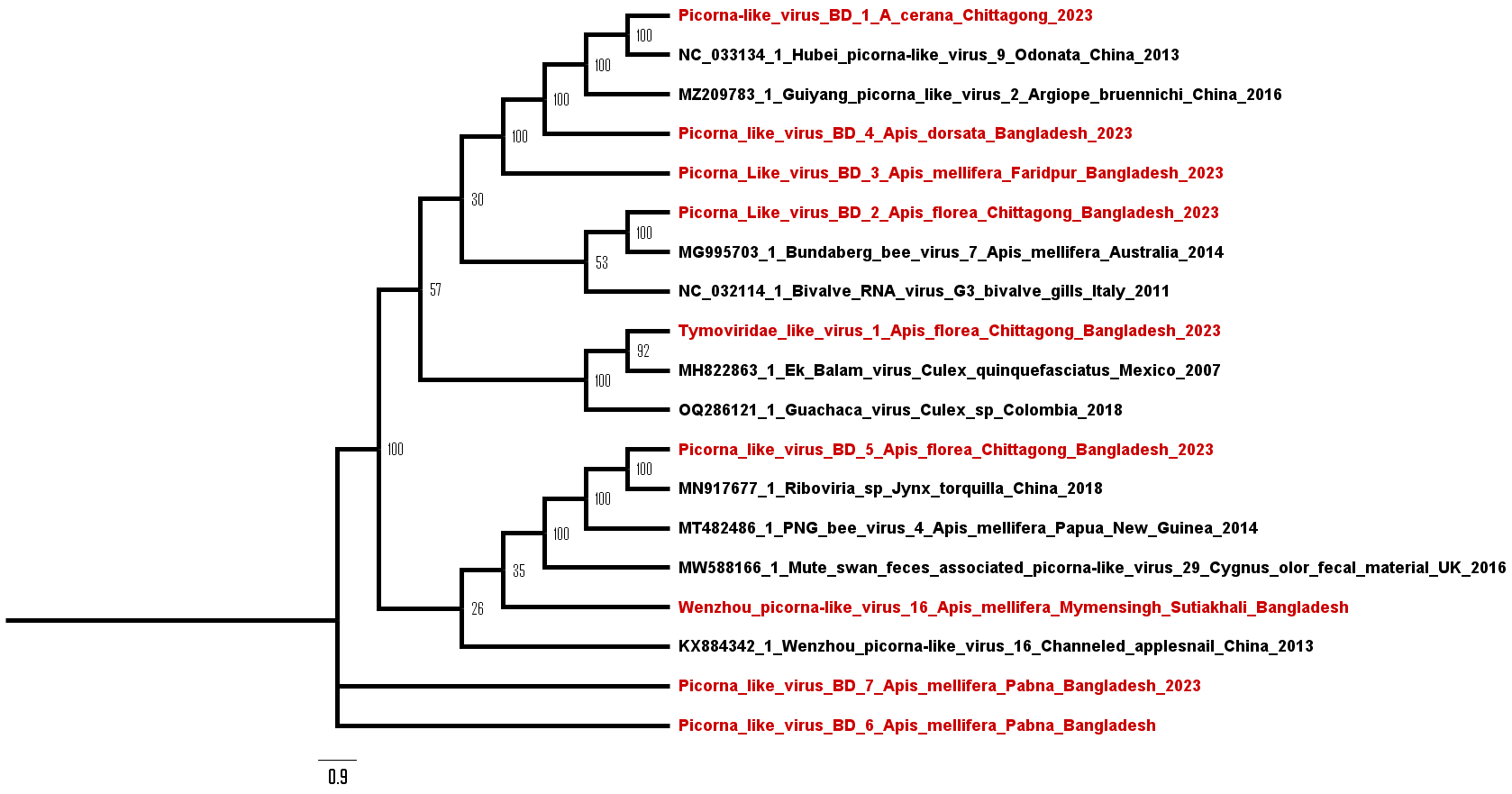


Supplementary figure 2. Phylogenetic analyses of unclassified of insect viruses in bee species in Bangladesh.
