# Supplementary material for: Insights into the viral landscape of the western honey bee and native bees in Bangladesh
Source: Microbiol Spectr. 2025 Nov 11;13(12):e01971-25. doi: 10.1128/spectrum.01971-25 (PMC12671125; doi:10.1128/spectrum.01971-25)
Supplement: Supplemental tables — Tables S1 to S3. [file spectrum.01971-25-s0004.docx]

**Supplementary Table 1: Prevalence and abundance of viral families in bee species in Bangladesh**

| **Family** | **Prevalence** | **Abundance (RPM)** | **Prevalence Rank** | **Abundance Rank** |
| --- | --- | --- | --- | --- |
| Dicistroviridae | 0.85714286 | 7718.25747 | 1 | 2 |
| Iflaviridae | 0.71428571 | 95098.5502 | 2 | 1 |
| Sinhaliviridae | 0.35714286 | 1062.95439 | 4 | 3 |
| Secoviridae | 0.21428571 | 118.710957 | 5 | 4 |
| Fusaviridae | 0.07142857 | 59.7292223 | 7 | 5 |
| Marnaviridae | 0.07142857 | 6.33537346 | 7 | 7 |
| Alphaflexiviridae | 0.07142857 | 5.29149405 | 7 | 8 |
| Unclassified | 0.42857143 | 43.9509934 | 3 | 6 |

Supplemeatary Table 2 **: Prevalence and abundance of insect viruses in bee species in Bangladesh**

| **Virus** | **Prevalence** | **Abundance** | **Prevalence_Rank** | **Abundance_Rank** |
| --- | --- | --- | --- | --- |
| BQCV | 0.5 | 2858.35328 | 2.5 | 4 |
| Bee_Iflavirus_BD_6 | 0.07142857 | 4.2490909 | 18.5 | 15 |
| Bee_associated_cripavirus_1 | 0.5 | 3060.31924 | 2.5 | 3 |
| Bee_dicistrovirus_10_BD | 0.07142857 | 1.2518612 | 18.5 | 25 |
| Bee_dicistrovirus_8_BD | 0.07142857 | 7.70248805 | 18.5 | 14 |
| Bee_dicistrovirus_9_BD | 0.07142857 | 1.16960992 | 18.5 | 26 |
| Bee_dicistrovirus_BD_1 | 0.42857143 | 1568.25331 | 4.5 | 5 |
| Bee_dicistrovirus_BD_3 | 0.21428571 | 9.24083399 | 10 | 13 |
| Bee_dicistrovirus_BD_4 | 0.07142857 | 21.637972 | 18.5 | 12 |
| Bee_dicistrovirus_BD_7 | 0.07142857 | 2.36097021 | 18.5 | 21 |
| Bee_iflavirus_BD_2 | 0.07142857 | 1.53746258 | 18.5 | 24 |
| DWV_A | 0.57142857 | 17090.3804 | 1 | 2 |
| DWV_B | 0.28571429 | 127.503804 | 7.5 | 9 |
| LSV_3 | 0.28571429 | 232.435757 | 7.5 | 7 |
| LSV_4 | 0.28571429 | 772.914518 | 7.5 | 6 |
| LSV_SA2 | 0.07142857 | 57.6041202 | 18.5 | 10 |
| Picorna_like_virus_BD_1 | 0.07142857 | 2.15488192 | 18.5 | 23 |
| Picorna_like_virus_BD_2 | 0.07142857 | 2.7906205 | 18.5 | 18 |
| Picorna_like_virus_BD_3 | 0.07142857 | 29.7796657 | 18.5 | 11 |
| Picorna_like_virus_BD_4 | 0.07142857 | 4.06025608 | 18.5 | 16 |
| Picorna_like_virus_BD_5 | 0.07142857 | 2.53193837 | 18.5 | 20 |
| Planococcus_ficus_associated_dicistrovirus_1 | 0.28571429 | 187.967906 | 7.5 | 8 |
| Sacbrood_virus | 0.42857143 | 77867.0752 | 4.5 | 1 |
| Varroa_destructor_virus_2 | 0.07142857 | 2.9851447 | 18.5 | 17 |
| Victoria_bee_virus_2 | 0.07142857 | 2.25568396 | 18.5 | 22 |
| Xiangshan_picorna_like_virus_2 | 0.07142857 | 2.56349124 | 18.5 | 19 |

Supplementary Table 3 **: Alpha diversity analysis of insect viruses in bee species in Bangladesh**

| **Library** | **Group** | **Shannon_Index** | **Simpson_Index** | **Species_Richness** |
| --- | --- | --- | --- | --- |
| A_mellifera_Mymensingh_S | Western_honeybee | 0.630534 | 0.325032 | 8 |
| A_mellifera_Mymensingh_M | Western_honeybee | 0.668398 | 0.346067 | 6 |
| A_mellifera_Pabna | Western_honeybee | 0.158946 | 0.071635 | 2 |
| A_mellifera_Faridpur | Western_honeybee | 0.756764 | 0.341912 | 8 |
| A_mellifera_Dhaka | Western_honeybee | 0.848841 | 0.515239 | 6 |
| A_mellifera_Shatkhira | Western_honeybee | 0.110863 | 0.039616 | 5 |
| A_melifera_Tangail | Western_honeybee | 1.595792 | 0.788956 | 8 |
| A_mellifera_Tangail_1 | Western_honeybee | 0.1554 | 0.058685 | 4 |
| Apis_cerana_Faridpur | Native_honeybee | 1.051199 | 0.63653 | 3 |
| Apis_cerana_Chittagong | Native_honeybee | 1.475899 | 0.619181 | 9 |
| A_dorsata_Chittagong | Native_honeybee | 1.447653 | 0.693319 | 7 |
| Apis_florea_Chittagong | Native_honeybee | 1.034038 | 0.620921 | 3 |
